# Dataset concerning effects of stevia (*Stevia rebaudiana* bertoni), amlodipine, losartan, and valsartan on water consumption, blood glucose and heart tissue in gentamycin-induced nephrotoxicity in the rat model

**DOI:** 10.1016/j.dib.2020.105965

**Published:** 2020-07-03

**Authors:** Farhana Rizwan, Saquiba Yesmine, Ishtiaque Ahmed Chowdhury, Sultana Gulshana Banu, Tapan Kumar Chatterjee

**Affiliations:** a Department of Pharmaceutical Technology, Jadavpur University, Kolkata-700032, India; b Department of Pharmacy, East West University, Aftabnagar, Dhaka-1212, Bangladesh; c Department of Pharmacy, Jahangirnagar University, Savar, Dhaka, Bangladesh; d Department of Pathology, Bangabandhu Sheikh Mujib Medical University, Shahbag, Dhaka, Bangladesh; e Department of Pharmaceutical Science and Technology, JIS University, Agarpara, Kolkata-700109, West Bengal, India

**Keywords:** Angiotensin-ii type-1 receptor (at1) blockers, Calcium (ca2+) channel blocker, Gentamycin-induced nephrotoxicity, stevia

## Abstract

This dataset indicates the effect of stevia (*Stevia rebaudiana* Bertoni); angiotensin-II type-1 receptor (AT_1_) blockers, losartan and valsartan; and a calcium (Ca^2+^) channel blocker, amlodipine; on water consumption, fasting blood glucose, and cardiac histology in gentamycin-induced nephrotoxic rat model. Six groups of male Sprague-Dawley rats were selected as sham control group, gentamycin-induced nephrotoxic disease control group; gentamycin-induced disease control groups treated with stevia (200 mg/kg/day); amlodipine (4 mg/kg/day); losartan (15 mg/kg/day) and valsartan (5 mg/kg/day) respectively. Fasting blood glucose level and water consumption were recorded daily for the first week and then weekly for the rest of treatment period. Serum creatinine, blood urea, total protein and lipid profile were determined. Histological examination of the heart tissue was assessed to find out any alteration of cardiac muscle tissue following gentamycin-induced nephrotoxicity. This article provides additional data collected from the same animals previously reported [1] .

**Specifications Table**

**Table:** 

Subject	Pharmacology and Toxicology
Specific subject area	Vascular pharmacology and toxicology
Type of data	Table and figures
How data were acquired	ACCU-CHEK Active (UK). Dimension RxL Max integrated Chemistry's system (USA) automated biochemistry analyzer. Rotary microtome (Thermo Fisher Scientific, Model: HM 325, UK). ZEISS Axio Microscope, Germany (Camera: Leica) at 10X, 20X and 40X magnification.
Data format	Raw and analyzed.
Parameters of data collection	Water consumption and fasting blood glucose levels were measured during the experimental period. Parameters measured for heart tissue were- congestion in blood vessels, thick walled blood vessels, deposition of inflammatory cells, edema, thick endocardium layer with fibrosis, heart chamber packed with RBC and congestion, focal mild edema with inflammation, myxoid changes in the heart muscle.
Description of data collection	Fasting blood glucose levels were measured in overnight fasted rats (12 h) every week during the 30 days experimental period. Water consumption for each rat per day was measured daily for the first week then weekly for the rest of the treatment period. Histology of the heart tissue and biochemical data were analyzed to examine the effect of stevia, amlodipine, losartan and valsartan treatment on these parameters in gentamycin-induced nephrotoxicity. Therefore, tissue and blood samples were taken from both the treatment as well as untreated nephrotoxicity groups at the end of the treatment period. Nephrotoxicity was induced by gentamycin [1]. Tissues from the heart were fixed at 10% buffered formalin solution, embedded with paraffin wax, sectioned about 4–5 μm thickness using a rotary microtome. Finally, Hematoxylin and Eosin (H&E) staining protocols was followed [2] and photomicrographs were taken.
Data source location	Division of Pharmacology of Jadavpur University, Kolkata, India.
Data accessibility	Data is within the article.
Related research article	F. Rizwan, S. Yesmine, S. G. Banu, I. A. Chowdhury, M. R. Hasan, T. K. Chatterjee. Renoprotective effects of stevia (*Stevia rebaudiana* Bertoni), amlodipine, valsartan, and losartan in gentamycin-induced nephrotoxicity in the rat model: Biochemical, hematological and histological approaches. Toxicology Reports. 6 (2019) 683–691.
	DOI: http://doi.org/10.1016/j.toxrep.2019.07.003 eCollection 2019.

**Value of the data**

• The dataset provides information on water consumption, fasting blood glucose level and cardiac histology in gentamycin-induced chronic kidney disease in rodent model.
• The dataset gives evidence supporting heart tissue damage in chronic kidney disease.
• The data compare the effect of stevia, angiotensin receptor blockers (ARBs) and calcium (Ca^2+^) channel blockers (CCBs) on heart tissue and biochemical parameters in gentamycin-induced nephrotoxicity in rat model.
• This dataset may be useful for cardiovascular pharmacologists and other researchers in evaluating suitable treatment options for hypertension in chronic kidney disease patients with cardio-protective effects.
• The data will be helpful to further research on end organ damage in chronic kidney disease and establish the beneficial role of stevia along with the most modern treatments, i.e., ARBs and calcium channel blockers.

## 1 Data description

This article presents the data of water consumption, fasting blood glucose and histology of heart muscle to demonstrate the effects of stevia, amlodipine, valsartan, and losartan in gentamycin-induced nephrotoxicity in the rat model. Table 1.0 (A) and Table 1.0 (B) show the comparison of weekly water consumption capacity and repeated measures ANOVA followed by pairwise comparison of the weekly water consumption for the 30 days treatment period respectively. Table 2.0 (A) and Table 2.0 (B) present the pattern of day to day water consumption and repeated measures ANOVA followed by pairwise comparison for the daily water consumption recorded for the first week of the treatment between the treatment groups of gentamycin-induced nephrotoxic rats respectively. An evaluation of raw data distribution (%) for biochemical parameters is produced and presented in Table 3.0. Table 4.0 (A) and Table 4.0 (B) demonstrate the comparison of fasting blood glucose (FBG) and repeated measures ANOVA followed by pairwise comparison for the FBG between the treatment groups of gentamycin-induced nephrotoxic rats during the treatment period. Table 5.0 represents toxicological investigation based on histological scoring of heart tissue of the experimental rat model. Fig. 1.0 represents the photomicrographs of heart sections of rats at the end of treatment period at different magnification (H&E stain, X10, X20, X40).

**Table 1:** (A) Comparison of weekly water consumption capacity between different treatment groups of gentamycin-induced nephrotoxic rats.

Treatment groups	Week-0	Week-1	Week-2	Week-3	Week-4
**STD**	55.52 (±1.69)	62.28 (±4.08)	52.06 (±7.95)	56.19 (±2.78)	56.52 (±3.60)
**CON**	37.40 (±2.19) *	55.88 (±1.66)	52.10 (±2.79)	55.54 (±1.21)	56.52 (±0.99)
**STV**	55.52 (±1.99)	62.28 (±3.93)	52.06 (±2.48)	56.18 (±2.86)	56.52 (±2.74)
**AML**	44.23 (±11.26)	51.78 (±13.35)	43.12 (±11.47)	45.20 (±11.67)	43.74 (±11.38)
**LOS**	43.10 (±10.86)	52.24 (±13.41)	48.74 (±12.29)	49.00 (±12.28)	49.50 (±12.43)
**VAS**	44.50 (±11.19)	50.22 (±12.87)	42.62 (±11.24)	43.98 (±11.22)	43.86 (±11.33)

(B) Repeated measures ANOVA followed by pairwise comparison for the weekly water consumption between different treatment groups of gentamycin-induced nephrotoxic rats						
WEEK	WEEK	Mean Difference	Std. Error	Sig.	95% Confidence Interval for Difference	
					Lower Bound	Upper Bound
1	2	−10.077*	1.439	.000	−14.491	−5.663
	3	−1.933	2.145	1.000	−8.512	4.646
	4	−4.783*	1.513	.040	−9.422	−0.144
	5	−4.888	1.779	.108	−10.344	.568
2	1	10.077*	1.439	.000	5.663	14.491
	3	8.144*	1.349	.000	4.008	12.281
	4	5.294*	.970	.000	2.319	8.269
	5	5.189*	1.467	.015	.690	9.688
3	1	1.933	2.145	1.000	−4.646	8.512
	2	−8.144*	1.349	.000	−12.281	−4.008
	4	−2.851	1.323	.407	−6.909	1.208
	5	−2.956	1.931	1.000	−8.878	2.967
4	1	4.783*	1.513	.040	.144	9.422
	2	−5.294*	.970	.000	−8.269	−2.319
	3	2.851	1.323	.407	−1.208	6.909
	5	−0.105	.782	1.000	−2.504	2.294
5	1	4.888	1.779	.108	−0.568	10.344
	2	−5.189*	1.467	.015	−9.688	−0.690
	3	2.956	1.931	1.000	−2.967	8.878
	4	.105	.782	1.000	−2.294	2.504

**Table 2:** (A) Day to day variation of water consumption for the first week of the treatment period between the different treatment groups of gentamycin-induced nephrotoxic rats.

Treatment groups	Day-1	Day-2	Day-3	Day-4	Day-5	Day-6	Day-7
**STD**	55.52 (±1.70)	56.30 (±2.55)	57.20 (±6.34)	67.66 (±3.24)	59.76 (±4.14)	64.86 (±3.24)	62.28 (±4.08)
**CON**	37.40 (±2.19) *	47.35 (±0.98) *	49.90 (±0.75)	56.72 (±1.01) *	50.24 (±2.76)	54.90 (±1.41)	55.88 (±1.65)
**STV**	55.52 (±1.99)	56.52 (±3.17)	57.96 (±6.15)	69.62 (±3.35)	61.82 (±5.18)	67.44 (±2.90)	62.28 (±3.93)
**AML**	44.22 (±11.25)	46.66 (±11.99)	48.16 (±12.53)	53.58 (±13.65)	53.94 (±14.02)	54.28 (±14.03)	51.78 (±13.35)
**LOS**	43.10 (±10.86)	45.36 (±11.37)	47.12 (±11.91)	48.14 (±12.45)	48.16 (±12.43)	50.27 (±12.94)	52.24 (±13.41)
**VAS**	44.50 (±11.19)	46.18 (±11.91)	46.56 (±13.05)	56.10 (±14.13)	49.88 (±13.15)	55.54 (±14.03)	50.22 (±12.87)

(B) Repeated measures ANOVA followed by pairwise comparison for the day to day variation of water consumption between the different treatment groups of gentamycin-induced nephrotoxic rats							
DAY	DAY	Mean Difference	Std. Error	Sig.	95% Confidence Interval for Difference		
					Lower Bound	Upper Bound	
1	2	−3.353	1.316	.359	−7.782	1.076	
	3	−4.933	2.005	.437	−11.681	1.815	
	4	−13.252*	1.472	.000	−18.206	−8.298	
	5	−8.063*	1.757	.002	−13.978	−2.148	
	6	−12.414*	1.369	.000	−17.021	−7.807	
	7	−10.078*	1.439	.000	−14.922	−5.233	
2	1	3.353	1.316	.359	−1.076	7.782	
	3	−1.580	1.101	1.000	−5.286	2.125	
	4	−9.899*	1.009	.000	−13.295	−6.503	
	5	−4.710*	1.077	.004	−8.335	−1.085	
	6	−9.061*	.866	.000	−11.977	−6.145	
	7	−6.725*	1.197	.000	−10.755	−2.694	
3	1	4.933	2.005	.437	−1.815	11.681	
	2	1.580	1.101	1.000	−2.125	5.286	
	4	−8.319*	1.557	.000	−13.560	−3.077	
	5	−3.130*	.921	.046	−6.229	−0.030	
	6	−7.480*	1.409	.000	−12.224	−2.736	
	7	−5.144	1.538	.053	−10.320	.031	
4	1	13.252*	1.472	.000	8.298	18.206	
	2	9.899*	1.009	.000	6.503	13.295	
	3	8.319*	1.557	.000	3.077	13.560	
	5	5.189*	1.233	.006	1.039	9.339	
	6	.838	.694	1.000	−1.498	3.174	
	7	3.174	1.599	1.000	−2.207	8.555	
5	1	8.063*	1.757	.002	2.148	13.978	
	2	4.710*	1.077	.004	1.085	8.335	
	3	3.130*	.921	.046	.030	6.229	
	4	−5.189*	1.233	.006	−9.339	−1.039	
	6	−4.351*	1.000	.004	−7.717	−0.985	
	7	−2.015	1.545	1.000	−7.217	3.187	
6	1	12.414*	1.369	.000	7.807	17.021	
	2	9.061*	.866	.000	6.145	11.977	
	3	7.480*	1.409	.000	2.736	12.224	
	4	−0.838	.694	1.000	−3.174	1.498	
	5	4.351*	1.000	.004	.985	7.717	
	7	2.336	1.342	1.000	−2.182	6.854	
7	1	10.078*	1.439	.000	5.233	14.922	
	2	6.725*	1.197	.000	2.694	10.755	
	3	5.144	1.538	.053	−0.031	10.320	
	4	−3.174	1.599	1.000	−8.555	2.207	
	5	2.015	1.545	1.000	−3.187	7.217	
	6	−2.336	1.342	1.000	−6.854	2.182

**Table 3:** Distribution (%) of raw data of biochemical parameters of the experimental rat model.

Treatment groups	Se. Urea		Se. Creatinine		RBS		Total Protein		Se. Albumin		CHL		TG		HDL		LDL	
	Nor	Ab	Nor	Ab	Nor	Ab	Nor	Ab	Nor	Ab	Nor	Ab	Nor	Ab	Nor	Ab	Nor	Ab
**STD**	50	50	83.3	16.7	66.7	33.3	100	0	100	0	16.7	83.3	66.7	33.3	66.7	33.3	33.3	66.7
**CON**	100	0	100	0	100	0	100	0	100	0	100	0	100	0	100	0	100	0
**STV**	83.3	16.7	83.3	16.7	66.7	33.3	100	0	100	0	66.7	33.3	83.3	16.7	100	0	100	0
**AML**	50	50	66.7	33.3	50	50	100	0	100	0	50	50	83.3	16.7	66.7	33.3	50	50
**LOS**	0	100	50	50	33.3	66.7	83.3	16.7	100	0	16.7	83.3	83.3	16.7	100	0	33.3	66.7
**VAS**	66.7	33.3	100	0	83.3	16.7	100	0	83.3	16.7	66.7	33.3	66.7	33.3	66.7	33.3	100	0

**Table 4:** (A) Comparison of fasting blood glucose (FBG) between the treatment groups during the experimental period of gentamycin-induced nephrotoxic rats.

Treatment groups	Day-7	Day-14	Day-21	Day-28		
**STD**	11.26 (±0.37)	10.56 (±0.46)	10.66 (±0.34)	10.94 (±0.50)		
**CON**	7.28 (±0.24) *	7.44 (±0.30) *	7.42 (±0.25) *	7.72 (±0.38) *		
**STV**	9.96 (±0.69)	7.84 (±0.29) *	7.78 (±0.15) *	8.80 (±0.53)		
**AML**	9.72 (±0.31) *	8.40 (±0.37) *	8.84 (±0.54) *	8.70 (±0.39) *		
**LOS**	8.58 (±0.38) *	8.56 (±0.35) *	8.54 (±0.26) *	8.56 (±0.19) *		
**VAS**	9.54 (±0.28) *	8.90 (±0.56) *	9.12 (±0.62) *	9.22 (±0.38) *		

(B) Repeated measures ANOVA followed by pairwise comparison for fasting blood glucose (FBG) between the treatment groups during the experimental period of gentamycin-induced nephrotoxic rats						
WEEK	WEEK	Mean Difference	Std. Error	Sig.	95% Confidence Interval for Difference	
					Lower Bound	Upper Bound
1	2	.753*	.174	.001	.265	1.240
	3	.700*	.195	.006	.153	1.247
	4	.308	.147	.256	−0.102	.718
2	1	−0.753*	.174	.001	−1.240	−0.265
	3	−0.053	.104	1.000	−0.344	.239
	4	−0.444*	.147	.027	−0.854	−0.035
3	1	−0.700*	.195	.006	−1.247	−0.153
	2	.053	.104	1.000	−0.239	.344
	4	−0.392	.154	.094	−0.823	.039
4	1	−0.308	.147	.256	−0.718	.102
	2	.444*	.147	.027	.035	.854
	3	.392	.154	.094	−0.039	.823

**Table 5:** Toxicological investigation based on histological scoring of heart tissue of the experimental rat model.

**Histological Features of Heart (*N*** **=** **6)**	**CON**	**STD**	**STV**	**AML**	**LOS**	**VAS**
Normal Branching of Heart Muscle	4/4/4/4/4/4	0	4/4/4/4/4/4	0	0	0
Normal Muscle Tissue	4/4/4/4/2/2	1/1	4/4/4/4/4/4	0	1/1	1/1/1
Congestion in Blood Vessels	0	3/3/3/3/3/3	0	2/2/2/2/2/2	0	2/2/2/2/2/2
Thick Walled Blood Vessels	0	0	0	2/2/2/2/2/2	0	0
Deposition of Inflammatory Cells	0	0	0	0	3/3/3/3	0
edema	0	0	0	0	3/3/3/3	0
Thick Endocardium Layer with Fibrosis	0	0	0	0	2/2/2/2	0
Heart Chamber packed with RBC & Congestion	0	0	0	0	3/3/3/3/3/3	0
Focal mild edema with inflammation	0	0	0	0	2/2	0
Myxoid Changes in the Heart Muscle	0	0	0	0	0	3/3/3

**Fig. 1:**
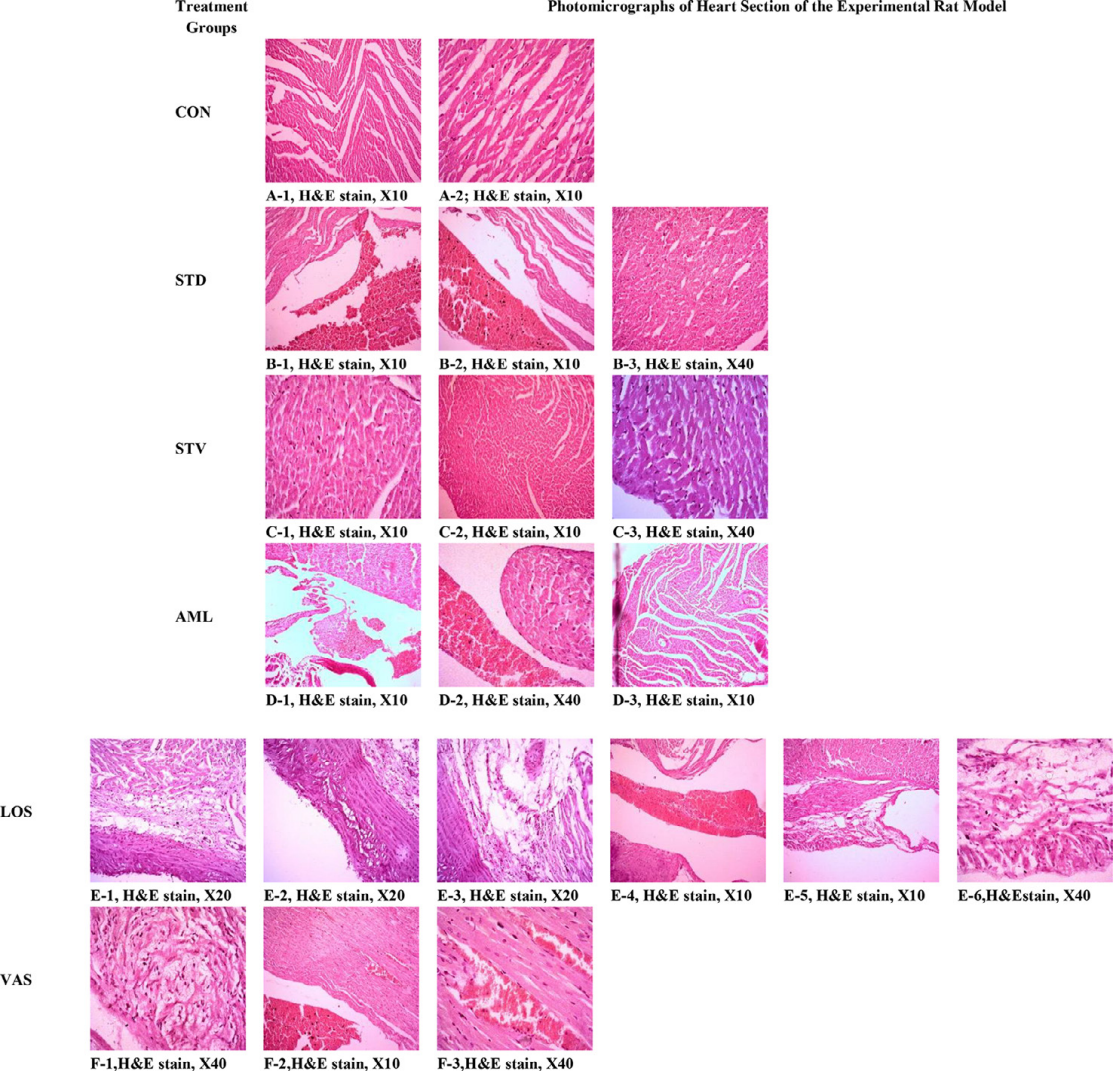
Photomicrographs of Heart sections of Rats at 30th days of the experimental period at Different Magnification (H&E stain, X10, X20, X40).

Here, values are expressed as Mean (±SEM). Paired sample-t-test was done to analyze the data. Sample size, *n* = 4–5. Treatment groups represent as CON=Healthy Control; STD=Gentamycin-induced disease control; AML=Gentamycin-induced disease control treated with amlodipine; LOS=Gentamycin-induced disease control treated with Losartan; VAS=Gentamycin-induced disease control treated with Valsartan; STV=Gentamycin-induced disease control treated with Stevia, respectively. A p-value less than 0.05 (*p* < .05) was considered as significant.

Here, a one-way repeated measure analysis of variance (ANOVA) followed by Pairwise comparison Test was conducted to evaluate the data. Multiple comparisons were adjusted by following Bonferroni test. *The mean difference is significant at the 0.05 level. Sample size, *n* = 4–5.

Here, values are expressed as Mean (±SEM). Paired sample-t-test was done to analyze the data. Sample size, *n* = 4–5. Treatment groups represent as CON=Healthy Control; STD=Gentamycin-induced disease control; AML=Gentamycin-induced disease control treated with amlodipine; LOS=Gentamycin-induced disease control treated with Losartan; VAS=Gentamycin-induced disease control treated with Valsartan; STV=Gentamycin-induced disease control treated with Stevia, respectively. A p-value less than 0.05 (*p* < .05) was considered as significant.

Here, a one-way repeated measure analysis of variance (ANOVA) followed by Pairwise comparison test was conducted to evaluate the data. Multiple comparisons were adjusted by following Bonferroni test. *The mean difference is significant at the 0.05 level. Sample size, *n* = 4–5.

Here, descriptive analysis was performed to analyze the data. Sample size, *n* = 6. Treatment groups represent as CON=Healthy Control; STD=Gentamycin-induced disease control; AML=Gentamycin-induced disease control treated with amlodipine; LOS=Gentamycin-induced disease control treated with Losartan; VAS=Gentamycin-induced disease control treated with Valsartan; STV=Gentamycin-induced disease control treated with Stevia, respectively. Se. Urea = Serum Urea; Se. Creatinine= Serum Creatinine; RBS= Random Blood Sugar; Se. Albumin= Serum Albumin; CHL= Cholesterol; TG=Triglyceride; HDL=High Density Lipoprotein; LDL=Low Density LipoProtein. Also, ‘Nor’ represents the normal values, and ‘Ab’ represents the abnormal values of the Dataset. Nor- Normal (the% values of the tests that fall within the limits of normal values demonstrated by the control group); Ab- Abnormal (the% values of the tests that are above the limits of normal values demonstrated by the control group).

Here, values are expressed as Mean (±SEM). Paired sample *t*-test was done to analyze the data. Sample size, *n* = 6. Treatment groups represent as CON=Healthy Control; STD=Gentamycin-induced disease control; AML=Gentamycin-induced disease control treated with amlodipine; LOS=Gentamycin-induced disease control treated with Losartan; VAS=Gentamycin-induced disease control treated with Valsartan; STV=Gentamycin-induced disease control treated with Stevia, respectively. A p-value less than 0.05 (*p* <0.05) was considered as significant.

Here, a one-way repeated measure analysis of variance (ANOVA) followed by Pairwise comparison test was conducted to evaluate the data. Multiple comparisons were adjusted by following Bonferroni test. *The mean difference is significant at the 0.05 level. Sample size, *n* = 6.

Here, multiparametric and semi-quantitative analysis were done for histological grading of heart tissue. Heart tissue damage was expressed as: no histological features are present =0; Histological features are present in up to 25% of the area examined =1; Histological features are present in >25% up to 50% of the area examined =2; Histological features are present in >50% up to 75% of the area examined =3; and Histological features are present in >75% of the area examined =4, based on the percentage of tissues affected in different treatment groups [3]. Treatment groups represent as CON=Healthy Control; STD=Gentamycin-induced disease control; AML=Gentamycin-induced disease control treated with amlodipine; LOS=Gentamycin-induced disease control treated with Losartan; VAS=Gentamycin-induced disease control treated with Valsartan; STV=Gentamycin-induced disease control treated with Stevia, respectively (*n* = 6 for each group).

In this Figure section, A (1 & 2), B-3, & C (1, 2, & 3) represents no change of the heart muscle of CON, STD, STV, & VAS groups of experimental rat; B (1, & 2), D (1, & 2), F (2, & 3) represents the congestion in the blood vessels of heart of STD, AML, & VAS groups of rat; D-3 represents the small thick walled blood vessels in the AML treated group of rat; E-1 represents the deposition of inflammatory cells and edema in the LOS treated group of rat. E-2 represents the thick endocardium layer with fibrosis in the LOS treated group of rat; E-3 represents edema in the LOS treated group of rat; E-4 represents the heart chamber, which is packed with red blood cells (RBC) and congestion has been observed in the LOS treated group of rat; E (5, & 6) represents the focal mild edema and inflammation in the LOS treated group of rat; F-1 represents mixed changes in the heart muscle of VAS treated group of rats. Treatment groups represent as CON=Healthy Control; STD=Gentamycin-induced disease control; AML=Gentamycin-induced disease control treated with amlodipine; LOS=Gentamycin-induced disease control treated with Losartan; VAS=Gentamycin-induced disease control treated with Valsartan; STV=Gentamycin-induced disease control treated with Stevia, respectively.

## 2 Experimental design, materials, and methods

Thirty-six male, eight weeks old Sprague-Dawley rats (180–200 gm) were taken for the experiment following an adaptation in the standard laboratory settings for one week prior to the study. All the requirements for experiments were maintained strictly, such as, room temperature of (22±3 °C), a humidity of 50±10% with a 12-hour light/dark cycles. Standard pellet diets, prepared by the Pharmacology Laboratory, Jahangirnagar University according to formula developed by the Bangladesh Council of Scientific and Industrial Research (BCSIR), Dhaka were used to feed the experimental animals and tap water were given to the animals ad libitum. The duration of the experiment was thirty (30) days [1]. The experimental animals were handled in absolute compliance and in accordance with the guidelines for the care and use of Laboratory Animals by the National Institute of Health (Ethic approval no: BBEC, JU/M 2019 (1)6). This project was carried out in the Division of Pharmacology of Jadavpur University, Kolkata, India and Jahangirnagar University, Dhaka, Bangladesh.

Gentamycin (100 mg/kg body weight/day; i. p. for 8 days) was used to induce nephrotoxicity to all animals but the sham controls and the rats were randomly divided into six groups (*n* = 6): healthy animals without nephrotoxicity as sham control (CON); Gentamycin-induced nephrotoxicity group as disease control (STD); gentamycin-induced disease control group treated with stevia (200 mg/kg/day; p.o.) (STV); gentamycin-induced disease control group treated with amlodipine (4 mg/kg/day; p.o.) (AML); gentamycin-induced disease control group treated with losartan (15 mg/kg/day; p.o.) (LOS); and gentamycin-induced disease control group treated with valsartan (5 mg/kg/day; p.o.) (VAS) respectively [4], [5], [6], [7], [8]. Treatments were started at 4 days before the gentamycin injection and carried out for thirty days [1]. Water intake was measured for each rat daily for the first week, then weekly for the rest of the treatment period. An overdose of sodium pentobarbital (60 mg/kg; i. p.) was used to euthanize the experimental animals after 30 days of experimental period [1].

Fasting blood glucose was measured after collecting blood samples from the tail vein of the overnight (12 h) fasted rats using ACCU-CHEK Active (UK), every week during the treatment period [1]. For the analysis of biochemical parameters, blood sample was collected from the posterior vena cava of each animal. To separate serum, the blood samples were allowed to clot and centrifuged at 3000 rpm for 15 min (MSE minor, England). Then the collected serum samples were stored in −80 °C for further analysis. Blood urea, serum creatinine, serum albumin, total protein, fasting blood glucose (FBG), random blood sugar (RBS), total cholesterol (TC), triglycerides (TG), low density lipoprotein (LDL), and high-density lipoprotein (HDL) were examined by using Dimension RxL Max integrated Chemistry's system (USA) automated biochemistry analyzer [1]. Plasma total protein analysis was done using the prism of a refractometer (Atago T2-NE, Japan) and result was measured in the concentration value of the scale.

The heart tissue was prepared for the examination of gross lesions of the experimental animals. For the fixation of the heart tissue, 10% buffered formalin solution was used. The fixation process was done in 48-h duration and embedded with paraffin wax. The samples were trimmed about 4–5 μm thickness of tissue sections using a sectioning rotary microtome (Thermo Fisher Scientific, Model: HM 325, UK), and sectioned tissues were kept directly into the water bath (45 °C), and then mounting was done. The mounted glass slides were preserved on a hot plate (54 °C) for the whole night. Finally, Hematoxylin and Eosin (H&E) staining protocol was followed for mounting slides [1], [2]. Photomicrographs were taken with ZEISS Axio Microscope, Germany (Camera: Leica) at 10X, 20X and 40X magnification.

A blind manner process was followed to identify the lesions of heart tissue and the degree of the lesion was scored using a multiparametric and semi-quantitative analysis to measure the intensity of histological changes observed [9], [10]. Normal branching of heart muscle, normal muscle tissue, congestion in blood vessels, thick walled blood vessels, deposition of inflammatory cells, edema, thick endocardium layer with fibrosis, heart chamber packed with red blood cells (RBC) and congestion, focal mild edema with inflammation, myxoid changes in the heart muscle were examined and were graded as: zero (0), which represents the absence of histopathological or change in histological features; Grade one (1) is applied for the changes in histological features that are present in up to 25% of area examined; Grade two (2) is used for the changes in histological features in >25% up to 50% of area examined; Grade three (3) is used to assess the changes in histological features that are present in >50% up to 75% of area examined; and Grade four (4) represents the changes in histological features are found in >75% of area examined, respectively [2].

Statistical analysis was performed using the SPSS software (Statistical Package for the Social Sciences, version 23.0, SPSS Inc, Chicago, III, USA). Data were analyzed to calculate the Standard Error Mean (±SEM) of biochemical parameters. Paired sample *t*-test and repeated measures ANOVA followed by pairwise comparison test were done to compare the weekly water consumption capacity, day to day variation of water consumption, and the fasting blood glucose (FBG) level of the gentamycin-induced nephrotoxic rat model and the treatment groups. Multiparametric and semi-quantitative analysis were followed for histological scoring of heart tissues.

## Declaration of Competing Interest

No competing interests have been found.

## Appendix Supplementary materials

Image, application 1

Image, application 2

Image, application 3
